# Advancements and Prospects in Cathode Materials for Aqueous Zinc-Ion Batteries: Mechanisms, Challenges and Modification Strategies

**DOI:** 10.3390/molecules30204143

**Published:** 2025-10-21

**Authors:** Yuewen Gong, Miao Jia, Qiong Yuan, Biao Yang

**Affiliations:** 1 Department of Material Science and Engineering, Beijing Technology and Business University, Beijing 100048, China; 18132084395@163.com; 2 College of Materials and Chemical Engineering, Zhengzhou University of Technology, Zhengzhou 450044, China; 20231006@zzut.edu.cn

**Keywords:** aqueous zinc-ion batteries (AZIBs), cathode materials, layered metal oxides, Prussian blue analogs, organic polymer materials

## Abstract

Owing to the inherent safety, environmental friendliness, and high theoretical capacity (820 mAh g^−1^) of zinc metal, aqueous zinc-ion batteries (AZIBs) have emerged as up-and-coming alternatives to organic lithium-ion batteries. However, the insufficient electrochemically active sites, poor structural stability, and severe interfacial side reactions of cathode materials have always been key challenges, restricting battery gravimetric energy density and cycling stability. This article systematically reviews current mainstream AZIB cathode material systems, encompassing layered manganese- and vanadium-based metal oxides, Prussian blue analogs, and emerging organic polymers. It focuses on analyzing the energy storage mechanisms of different material systems and their structural evolution during Zn^2+^ (de)intercalation. Furthermore, mechanisms of innovative strategies for improving cathodes are thoroughly examined here, such as nanostructure engineering, lattice doping control, and surface coating modification, to address common issues like structural degradation, manganese/vanadium dissolution, and interface passivation. Finally, this article proposes future research directions: utilizing multi-scale in situ characterization to elucidate actual reaction pathways, constructing artificial interface layers to suppress side reactions, and optimizing full-cell design. This review provides a new perspective for developing practical AZIBs with high specific energy and long lifespans.

## 1. Introduction

Driven by the global energy transition, new energy storage technologies that are safe, resource-sustainable, and environmentally compatible need to be developed urgently. Lithium-ion batteries (LIBs) dominate the market of portable electronics and electric vehicles due to their high energy density and long cycle life. However, the scarcity of lithium resources, their high costs, and the safety risks associated with organic electrolytes have spurred the exploration of alternative technologies. Recently, zinc-ion batteries (ZIBs) have garnered significant research and industrial interest due to their unique advantages. As a strategic resource with a crustal abundance of 75 ppm (3.75 times that of lithium), zinc exhibits distinctive electrochemical properties, including a lithium-like standard reduction potential (−0.76 V vs. SHE) alongside excellent electronic/ionic conductivity. Crucially, Zn offers a high theoretical capacity (820 mAh g^−1^), thus offering a high volumetric energy density (5855 mAh cm^−3^) for ZIBs [1,2,3,4,5]. These traits establish physicochemical foundations for safe ZIBs. Consequently, diverse systems have emerged, including Zn−Mn [6,7], Zn−Ag [8,9], Zn−Ni [10,11], Zn−air [12,13], and other ZIBs [14,15]. Among these, aqueous zinc-ion batteries (AZIBs) utilize aqueous electrolytes, offering advantages such as low cost, enhanced safety, environmental friendliness, stability, and high ionic conductivity [16,17,18,19], which has garnered extensive research attention.

Aqueous zinc-ion batteries (AZIBs) fundamentally comprise a zinc metal or zinc-based compound anode, a zinc-ion conductive electrolyte, a separator, and a cathode. Cathode materials have been attracting significant attention due to their chemical diversity and performance-defining role. Ideal cathode materials must exhibit high reversible specific capacity, excellent cycling stability, high intrinsic electronic conductivity, and rapid zinc-ion diffusion kinetics, alongside environmental compatibility and economic viability. Consequently, developing high-performance cathodes is crucial for the commercialization of AZIBs.

AZIB cathode materials primarily include five categories: manganese (Mn)-based compounds [20,21,22,23,24], vanadium (V)-based oxides/vanadates [25,26,27], Prussian blue analogs [28,29,30], organic compounds [31,32,33,34,35], and emerging materials [36,37,38,39]. While each one demonstrates unique merits, significant technological bottlenecks persist. Manganese-based compounds are promising candidates due to their multivalent states (Mn^2+^/Mn^3+^/Mn^4+^/Mn^7+^), moderate operational voltage, high theoretical capacity, and structural stability. However, there exist critical challenges, including Mn dissolution-induced capacity decay and irreversible phase transition-induced voltage hysteresis. V-based materials exhibit stable frameworks, exceptional cycling durability, and high reversible capacity; however, they suffer from complex multi-electron reaction mechanisms, low operational voltage plateaus, and V dissolution during extended cycling. Prussian blue analogs attract attention due to their open frameworks, facile synthesis, and eco-friendliness; however, there is an urgent need to solve their inherent low specific capacity, limited potential windows, and poor cycling stability. Organic compounds, as a kind of emerging cathode, offer high theoretical capacity, tunable molecular structures, synthetic controllability, and environmental compatibility; however, they commonly exhibit low working voltages, poor structural integrity, and sluggish ion transport kinetics in practical applications.

In recent years, considerable advances have been achieved in the development of high-performance cathode materials for aqueous zinc-ion batteries. Facing frontiers of the domain, this article systematically investigates the structure–performance relationship, divergent energy storage mechanisms, and the characteristic electrochemical behaviors of the representative cathode materials as mentioned above. It critically reviews their application potential, technical bottlenecks, and the targeted modification strategies proposed in contemporary research. Subsequent sections will categorically discuss the characteristics, classification, research progress, and direction of each cathode material.

## 2. Cathode Material of AZIBs

### 2.1. Mn-Based Cathodes

Among all cathode materials, Mn-based oxides present the advantages of low cost, high theoretical specific capacity, and abundant reserves, rendering them one of the most commercially promising candidates. The multivalent redox characteristics of these materials (involving Mn^2+^/Mn^3+^/Mn^4+^ transitions) prompt flexible electrochemical energy storage mechanisms [40], enabling formation of multicomponent systems including MnO_2_ (layered/tunnel structures) [41,42,43], MnO/Mn_2_O_3_ (orthorhombic phase) [44,45,46], Mn_3_O_4_ (spinel phase) [47,48], and ZnMn_2_O_4_ (spinel composite) [49,50,51]. This diversity provides substantial design dimensions for optimizing ion diffusion pathways and enhancing structural stability.

Although Mn-based oxides exhibit significant cost and resource advantages, their multivalent characteristics often induce dynamic evolution of crystal structures (e.g., Jahn–Teller distortion). This phenomenon, coupled with persistent Mn dissolution and proton intercalation side reactions at the electrolyte/electrode interface [52], collectively results in the complex charge storage behavior of this system. The intricacy of such electrochemical-structural coupling causes sustained controversies regarding the energy storage mechanisms of Zn/MnO_x_ batteries [53,54]. Current mechanistic discussions primarily center on four models:

(1) Zn^2+^ Intercalation/Deintercalation Mechanism

**Figure 1:**
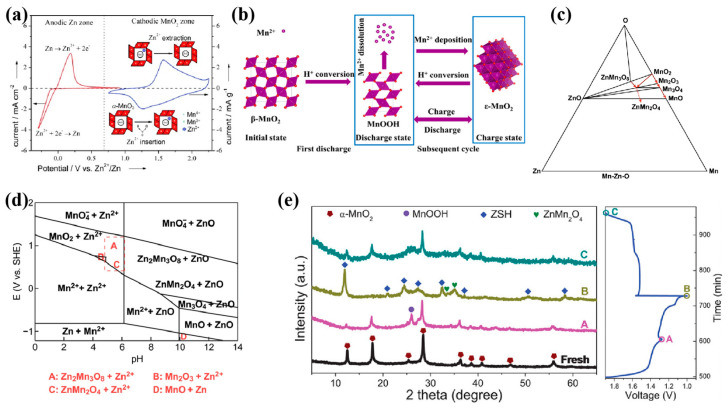
(**a**) Cyclic voltammogram of the zinc anode (red line) and the α-MnO_2_ cathode (blue line) at 2 mV s^−1^ in 0.1 mol L^−1^ Zn(NO_3_)_2_ aqueous electrolyte (pH 5.2). The plots show the anodic and cathodic processes of the zinc-ion battery, respectively [55]. (**b**) The reaction path of β-MnO_2_ during cycling [58]. (**c**) Zn–Mn–O diagram and (**d**) E-pH diagram of Zn–Mn–H_2_O system [64]. (**e**) Ex situ XRD patterns at different depths of the charge/discharge process of MnO_2_/rGO nanocomposite [66].

As an early foundational model, this mechanism was inspired by the similar ionic radii of Zn^2+^ and Li^+^ (0.74 Å vs. 0.76 Å). Xu et al. [55] first demonstrated in 2012 that in mild aqueous solutions of ZnSO_4_ and Zn(NO_3_)_2_, Zn^2+^ can reversibly intercalate into the α-MnO_2_ tunnel structure (Figure 1a), accompanying the formation of spinel-phase ZnMn_2_O_4_. The reaction proceeds as follows:

Cathode: 2α-MnO_2_ + Zn^2+^ + 2e^−^ ⇌ ZnMn_2_O_4_ (1)

Anode: Zn ⇌ Zn^2+^ + 2e^−^ (2)

(2) Phase-Transition-Dominated Transformation Mechanism

This model focuses on crystal structure reconstruction in MnO_2_ (e.g., MnO_2_ → ZnMn_2_O_4_ or MnOOH), accompanied by stepwise changes in Mn valence [56,57]. Liu et al. [58] synthesized β-MnO_2_ nanorods via hydrothermal synthesis, combining in situ XRD and ex situ SEM/XPS characterization to elucidate the β-MnO_2_ → MnOOH structural evolution pathway during electrochemical cycling (Figure 1b). The reaction proceeds as follows, concurrent with proton (H^+^) insertion-induced lattice distortion and dynamic electrolyte pH fluctuations:

Cathode: MnO_2_ + H^+^ + e^−^ ⇌ MnOOH; (3)

MnOOH + 3H^+^ + e^−^ ⇌ Mn^2+^ +2H_2_O (4)

4Zn^2+^ + SO_4_^2−^ +8H_2_O ⇌ Zn_4_SO_4_(OH)_6_·5H_2_O +6H^+^ (5)

Anode: Zn ⇌ Zn^2+^ + 2e^−^ (6)

(3) H^+^/Zn^2+^ Co-intercalation Mechanism

This model emphasizes the cooperative intercalation behavior of protons (H^+^) and Zn^2+^ in acidic electrolytes [59,60,61]. However, due to its smaller ionic radius and higher mobility than Zn^2+^, H^+^ preferentially intercalates into the MnO_2_ lattice, dominating initial-stage charge storage [62,63]. Huang et al. [64] systematically analyzed the synergistic energy storage pathway of α-MnO_2_ in ZnSO_4_/MnSO_4_ mixed electrolytes by constructing thermodynamic phase diagrams and potential-pH (*E*-pH) equilibrium diagrams of the Mn-Zn-O system (Figure 1c,d). The reaction proceeds as follows:

Cathode: 2MnO_2_ + xZn^2+^ + 2xe^−^ ⇌ Zn_x_Mn_2_O_4_ (7)

MnO_2_ + H^+^+ e^−^ ⇌ MnOOH (8)

2MnO_2_ + 2H^+^ + 2e^−^ ⇌ Mn_2_O_3_ + H_2_O (9)

4Zn^2+^+SO_4_^2−^ + 5H_2_O + 6OH^−^ ⇌ ZnSO_4_∙3Zn(OH)_2_∙5H_2_O (10)

Anode: Zn ⇌ Zn^2+^ + 2e^−^ (11)

(4) Dissolution/Deposition-Dominated Interface Mechanism

This mechanism involves the reversible migration of charge in the dissolution–deposition processes under electrochemical potential gradients [52]. The model quantifies the capacity contributions from dynamic dissolution–redeposition behavior at electrode/electrolyte interfaces. During cycling, H^+^/Zn^2+^ insertion/extraction occurs concurrently while partial MnO_2_ undergoes disproportionation, generating soluble Mn^2+^, which will co-deposit with Zn^2+^ to form Zn-Mn composite oxides (e.g., ZnMn_2_O_4_) [65]. Based on this, Wang et al. [66] engineered 1D ultralong MnO_2_ nanowire/2D reduced graphene oxide (rGO) nanosheet heterojunctions, confirming the energy storage mechanism of the MnO_2_ cathode involves consecutive H^+^/Zn^2+^ insertion/extraction and the reversible dissolution/deposition of zinc sulfate hydroxide hydrate (ZSH, ZnSO_4_[Zn(OH)_2_]_3_·xH_2_O). The first discharge plateau corresponds to H^+^ insertion forming MnOOH, while the second is associated with Zn^2+^ insertion and concomitant ZSH precipitation due to local OH^−^ accumulation; upon charging, ZSH dissolves and α-MnO_2_ is recovered, confirming a combined insertion–deposition mechanism. (Figure 1e). The reaction proceeds as follows:

Cathode: MnO_2_ + H^+^ + e^−^ ⇌ MnOOH (12)

2MnO_2_ + Zn^2+^ + 2e^−^ ⇌ ZnMn_2_O_4_ (13)

4Zn^2+^ + 6OH^−^ + SO_4_^2−^ + xH_2_O ⇌ ZnSO_4_[Zn(OH)_2_]_3_∙xH_2_O (14)

Anode: Zn ⇌ Zn^2+^ + 2e^−^ (15)

The diversity of Mn-based oxide cathode materials and the complexity of their energy storage mechanisms furnish extensive research dimensions for performance optimization. Based on this, from the perspective of the multivalent characteristics of Mn and its diverse crystallographic architectures, integrated with energy storage mechanisms, this section systematically examines the practical material design breakthroughs of MnO_2_ cathodes.

#### 2.1.1. MnO_2_

**Figure 2:**
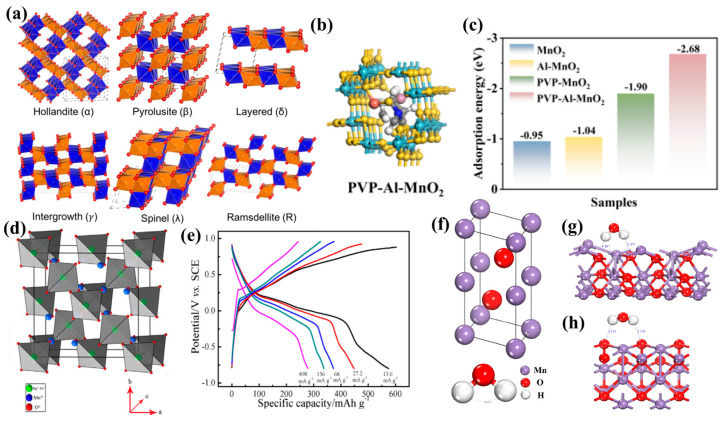
(**a**) MnO_2_ polymorphs [70]; (**b**) theoretical simulations, structural models of PVP-Al-MnO_2_, (**c**) PVP-Al-MnO_2_ relative adsorption energy (*E*_ads_) of Zn-ion absorbed [80]; (**d**) crystal structure of MMn_2_O_4_(M = Mg, Zn), (**e**) galvanostatic charge and discharge profiles in 0.5 mol dm^−3^ MgCl_2_ electrolyte [85]; (**f**) models of ε-MnO_2_ and water molecules, (**g**,**h**) geometrically optimized models of (110) and (102) planes [86].

Despite the significant chemical diversity in Mn-based cathode systems, MnO_2_ remains the predominant choice for AZIBs [67,68], due to its fundamental advantage stemming from the rich polymorphic structures formed through [MnO_6_] octahedral units connecting via edge- or corner-sharing, creating distinct topological frameworks including α-MnO_2_ (2 × 2 tunnel), β-MnO_2_ (1 × 1 tunnel), γ-MnO_2_ (disordered intergrowth), δ-MnO_2_ (layered), and λ-MnO_2_ (spinel) crystalline isomers (Figure 2a) [69,70,71]. Such structural diversity fundamentally arises from variations in octahedral connectivity: α-MnO_2_ forms 3D tunnel networks through double-chain corner-sharing, whereas δ-MnO_2_ achieves 2D layered frameworks via single-layer edge-sharing. These topological differences directly govern the dimensionalities and sizes of zinc-ion diffusion pathways, establishing the structural basis for regulating ion transport kinetics.

As the predominant cathode material for AZIBs, MnO_2_ offers a substantial theoretical capacity and voltage potential; however, it faces practical limitations due to its intrinsically low electronic conductivity (<10^−5^ S cm^−1^) and Jahn–Teller distortion-induced structural degradation. Recent advances have shown that defect engineering can be achieved by introducing vacancies through heteroatomic doping (metal/non-metal ion modulation), effectively modulating the electronic structures and relieving lattice strain, thereby synergistically enhancing energy storage performance of Zn [72,73,74]. For metal doping, Lian et al. [75] fabricated Ti-doped α-MnO_2_ nanowires (Ti-MnO_2_ NWs) via atomic layer deposition-enabled solid diffusion. Ti^4+^ substitution-induced lattice shrinkage, while the d^0^ electronic state of Ti^4+^ injected electrons into the conduction band of MnO_2_. This reduces the valence of Mn and promotes oxygen vacancy formation, thereby alleviating structural stress. Regarding non-metal doping, Zhang et al. [76] synthesized N-doped MnO_2_ hydrothermally using urea, where p-type N-doping elevated the Fermi level, significantly enhancing electronic conductivity and interfacial charge transfer. Furthermore, Chen et al. [77] engineered N/S-co-doped N-Mn_x_O_y_-S, where S-doping triggered a morphological reconstruction from rods to irregular polygons, increasing the specific surface area from 7.1 to 22.0 m^2^ g^−1^, thereby providing an expanded electrolyte penetration space and buffering volume changes. N/S-co-doping generated Mn-N-S active sites that elevated Zn^2+^ diffusion coefficients to 10^−6^~10^−5^ cm^2^ s^−1^. Meanwhile, sulfur/oxygen vacancies established dual defect channels that synergistically enhanced Zn^2+^ diffusion into Mn. The mixed Mn^2+^/Mn^3+^ valence states enabled continuous dual electron/ion transports, achieving 187 mAh g^−1^ at 1000 mA g^−1^ and retaining 103 mAh g^−1^ at 2000 mA g^−1^ with 90% capacity retention after more than 3000 cycles. Crucially, N/S-doping in N-Mn_x_O_y_-S not only enhanced conductivity but also improved interfacial kinetics through Mn-S/Mn-N bonding, effectively mitigating Mn dissolution. These studies establish that precise doping control—by modulating element type (metal/non-metal) and concentration gradients—not only reconstructs the local coordination of MnO_2_ at atomic scales, but also optimizes ion transport pathways at the mesoscopic level, ultimately achieving triple breakthroughs in intrinsic conductivity, structural stability, and reaction kinetics, thereby establishing a new paradigm for the rational design of high-stability Mn-based cathodes.

For a long time, researchers regarded α-MnO_2_ as an ideal cathode candidate for aqueous zinc-ion batteries (AZIBs) due to its unique tunnel structure. However, Mn dissolution and structural collapse during cycling cause capacity decay, severely limiting practical applications. Consequently, researchers employed nano-engineering, carbon compositing, and doping strategies to enhance the electrochemical performance of α-MnO_2_. In terms of nanostructure design, Li et al. [78] developed hierarchical α-MnO_2_@La_x_Mn_1−x_O_2-δ_ core–shell nanostructures (α-MnO_2_@LMO), where internal hollow nanotubes mechanically support external La_x_Mn_1−x_O_2-δ_ nanosheets to form porous, robust architectures. The α-MnO_2_@LMO cathode delivers an initial capacity of 240 mAh g^−1^ and maintains 115 mAh g^−1^ after 1500 cycles at 1 A g^−1^. Mechanistic analyses confirm that the core–shell structure enhances MnO_2_ stability, reduces Mn dissolution, accelerates Zn^2+^ transport kinetics, and suppresses irreversible ZnMn_2_O_4_ formation during H^+^/Zn^2+^ intercalation. For doping, Ren et al. [79] grew Mo-doped α-MnO_2_ (MMO) on carbon cloth (CC) as flexible cathodes. The pillar effect of Mo stabilizes tunnel structures while accelerating charge carrier diffusion. CC@MMO exhibits 80% capacity retention after 1392 cycles at 0.5 A g^−1^. Flexible devices retain 98.3% of their capacity under mechanical deformation at 2.5 mA cm^−2^, maintaining functionality after cutting or compression. Tian et al. [80] incorporated Al^3+^ and polyvinylpyrrolidone (PVP) into α-MnO_2_ tunnels (PVP-Al-MnO_2_) via organic-inorganic co-modification. Structural DFT analyses reveal Al^3+^/PVP co-intercalation reduces Zn^2+^ adsorption energy versus pristine α-MnO_2_ (Figure 2b,c), facilitating rapid Zn^2+^ diffusion and stable storage. The PVP-Al-MnO_2_ electrode achieves 306.8 mAh g^−1^ at 0.3 A g^−1^ and 93.1% retention after 2000 cycles at 1.0 A g^−1^. Beyond heteroatom doping, carbon nanomaterials could also enhance MnO_2_ cathodes by addressing slow charge transfer and cycling instability [81,82]. Islam et al. [83] utilized carbon-coated α-MnO_2_ to boost conductivity and suppress Mn dissolution. Minjie Shi et al. [84] designed 3D nanoflower-like GQDs@Zn_x_MnO_2_ composites by coupling graphene quantum dots (GQDs) with Zn-intercalated MnO_2_ nanosheets. GQD modification and Zn-embedding provide active sites and conductive media, enabling enhanced charge transfer. Therefore, the GQDs@Zn_x_MnO_2_ cathode exhibits exceptional Zn-storage capacity (403.6 mAh g^−1^) on account of fast kinetics and reversibility. The resulting AZIBs deliver high energy density (226.8 Wh kg^−1^), power density (650 W kg^−1^), and long-cycle performance, validating the unique advantage of carbon nanocomposites in balancing high energy density and cycling stability.

For Mn-based cathodes, crystal topology intrinsically governs the energy storage characteristics of MnO_2_ polymorphs. While tunnel-structured T-MnO_2_ (3 × 3 tunnels: 7.0 × 7.0 Å^2^) benefits ion transport and storage, residual impurity cations and crystalline water molecules in the tunnels impede ionic diffusion and storage. In addition, its complex synthesis process further limits applicability. Conversely, layered δ-MnO_2_ provides rapid Zn^2+^ migration through an ~0.7 nm interlayer spacing; however, it suffers from structural collapse due to inter-layer bond dissociation during the extended cycling, resulting in irreversible phase transition, substantial volume change, and Mn dissolution, which critically compromises the cycling stability. Spinel-type λ-MnO_2_ (Fd-3m space group) exhibits limited initial capacity due to inadequate ion transport channels. However, hierarchical pore architectures can be engineered via acid-etching strategies, as exemplified by the conversion of spinel LiMn_2_O_4_ to porous λ-MnO_2_ realized by Cao et al., achieving a discharge capacity of 545.6 mAh g^−1^ in 0.5 mol dm^−3^ MgCl_2_ electrolyte (Figure 2d,e) [85]. Densely packed ε-MnO_2_ suffers from low electrochemical activity and conductivity, which can be ameliorated through structural water intercalation and defect engineering. Zhang et al. [86] introduced structural water, nitrogen doping, and oxygen vacancies into ε-MnO_2_ via ball milling. Structural water on (102) and (110) planes reduces Zn^2+^-host electrostatic interactions, enhancing ion diffusion kinetics (Figure 2f–h), and nitrogen doping increases zinc-ion storage in oxygen vacancies. These studies demonstrate that the synergistic modulation of intrinsic defects and extrinsic dopants can overcome the “structure–dynamics–stability” trilemma of MnO_2_, establishing multidimensional optimization pathways for high-performance Mn-based cathodes.

#### 2.1.2. Other Mn-Based Materials/MnO_x_

MnO_x_ represents archetypal layered cathode materials, endowed with exceptional charge transfer characteristics because of unique 2D ion diffusion channels (interlayer spacing ≈ 0.72 nm) and dual active centers—interlayer adsorption sites and surface redox sites. This structural configuration enables high electronic conductivity and rapid ion diffusion kinetics. Crucially, MnO_x_ possesses synergistic capacity contributions from both single-electron (Mn^4+^/Mn^3+^) and two-electron (Mn^4+^/Mn^2+^) redox processes, transcending traditional single-electron reaction limitations. However, these multivalent reactions will intensify Jahn–Teller distortion and drive an irreversible phase transition. Consequently, it is necessary for performance optimization to implement interlayer pillar engineering (e.g., heteroatom intercalation) coupled with surface passivation strategies (e.g., carbon coating).

**Figure 3:**
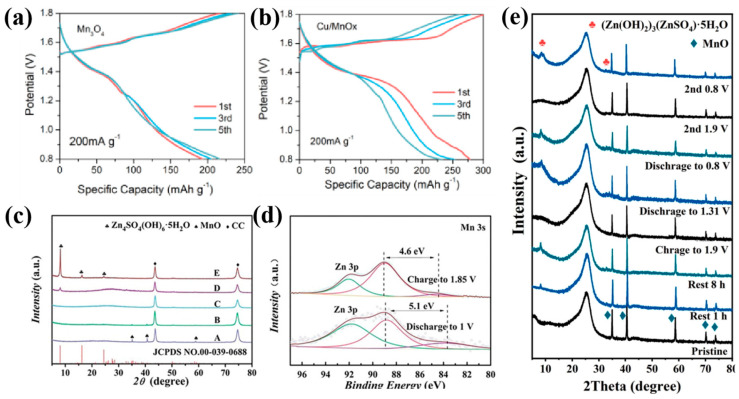
The charge and discharge curves for the 1st, 3rd, 5th cycles under 200 mA g^−1^ of (**a**) Mn_3_O_4_, (**b**) Cu/MnO_x_ [87]; (**c**) the corresponding ex situ XRD patterns in 2 M ZnSO_4_+0.2 M MnSO_4_ electrolyte, (**d**) XPS spectra of Mn 3s region at the charging and discharging states in 2 M ZnSO_4_+0.2 M MnSO_4_ electrolyte [88]; (**e**) ex situ XRD patterns of MnO at different states [92].

Han et al. [87] implemented Cu doping in MnO_x_, inducing localized charge redistribution and reducing the energy of oxygen vacancy formation. This atomic-level regulation establishes conductive pathways and expands electrochemical reaction surfaces, increasing the number of active sites and facilitating the conduction and storage of Zn^2+^. As shown in Figure 3a,b, Cu/MnO_x_ nanocomposites deliver 304.4 mAh g^−1^ at 0.2 A g^−1^ with a stable discharge plateau at 1.3 V, indicating relieved voltage polarization and reduced capacity decay. Zhou et al. [88] constructed Zn-ion battery cathodes using polydopamine-derived carbon-coated MnO nanoparticles (MnO/C-PDA), systematically investigating the effects of MnSO_4_ concentration (0.2 and 0.5 M) in ZnSO_4_ electrolytes. Optimal Mn^2+^ additives (0.2 M) can enhance electrode performance through optimizing interfacial kinetics, which achieves 295.4 mAh g^−1^ at 0.1 A g^−1^ with negligible degradation after 100 cycles. Ex situ characterization (Figure 3c,d) reveals a multistage energy storage mechanism involving the transformation from crystalline MnO to amorphous MnO_x_ during charging and H^+^/Zn^2+^ co-intercalation during discharging. Sun et al. [89] engineered core–shell Mn_3_O_4_@N-doped carbon nanorods (Mn_3_O_4_@NCNRs) via self-sacrificial templating. The N-doped carbon coating forms a continuous conductive network. Meanwhile, synergistic Zn^2+^/Mn^2+^ interactions enable exceptional cycling stability, achieving 280 mAh g^−1^ at 100 mA g^−1^ and maintaining 97 mAh g^−1^ after 700 cycles at 1000 mA g^−1^. Akmalia et al. [90] synthesized freestanding MnO_x_/N-doped carbon nanofiber cathodes via electrospinning. The MnO_x_–nanofiber interface promoted rapid charge transfer while suppressing active material detachment, thus achieving an unprecedented combination of rate and stability. Zhang et al. [91] designed defect-rich, non-stoichiometric MnO_x_ embedded in 3D carbon networks (MnO_x_/CN), which provided multidimensional ion-diffusion pathways and abundant electroactive sites for Zn^2+^ storage.

In addition, the reaction pathways of the MnO_x_ complex (such as intercalation, phase transition, and dissolution–deposition coexistence) and ambiguous capacity sources severely constrain the rational design of high-performance cathodes. To address this issue, Liu et al. [92] systematically compared the electrochemical activation behavior and reaction pathways of different manganese oxides (MnO_2_, Mn_2_O_3_, Mn_3_O_4_, and MnO), revealing the outstanding advantages of MnO in zinc-ion battery systems (Figure 3e). To simultaneously boost the poor intrinsic electronic conductivity and sluggish dissolution kinetics of MnO, the research team successfully constructed porous carbon-encapsulated MnO nanocomposites (MnO@PC). Utilizing the three-dimensional conductive network and multi-level mass transfer channels formed from porous carbon, side reactions were suppressed; simultaneously, the porous carbon matrix promoted electrolyte penetration and enhanced the adsorption of Mn^2+^ and Zn^2+^, resulting in sustained Zn_4_SO_4_⋅(OH)_6_⋅xH_2_O (ZSH) deposition and the stability of the reversible ZSH-assisted deposition/dissolution reaction. This design enables MnO@PC to achieve a breakthrough performance in pure ZnSO_4_ electrolyte, exemplified by a reversible capacity of 269 mAh g^−1^ at 0.1 A g^−1^ (89% of theoretical capacity) with 93% capacity retention after 120 cycles, while maintaining 75 mAh g^−1^ at 2.0 A g^−1^ and stable cycling over 2000 cycles. This study provides theoretical guidance and a technical paradigm for a high-stability AZIB cathode design by decoupling the intrinsic characteristics and the interface evolution laws of Mn-based oxides.

In summary, Mn-based oxides, as the core cathode materials for aqueous zinc-ion batteries, offer a high theoretical capacity and versatile storage mechanisms (intercalation, phase transition, synergistic effect of dissolution and deposition, etc.) due to their rich crystal structures (tunnel, layered, spinel, etc.) and multivalent redox activity (Mn^2+^/Mn^3+^/Mn^4+^). However, its practical application is limited by challenges including Mn dissolution, structural collapse (e.g., Jahn–Teller distortion), and poor intrinsic conductivity. To overcome these bottlenecks, researchers have substantially enhanced performance through multi-scale strategies including structural design (e.g., porous carbon encapsulation) for constructing efficient electron/ion transport networks, defect engineering (e.g., oxygen vacancies and N/S co-doping) to optimize charge distribution and reduce ion migration energy barriers, along with interface regulation (e.g., dynamic Mn^2+^ compensation and ZSH deposition stabilization) for suppressing side reactions. Additionally, battery performance can be enhanced through modification of the electrolyte [93]. Among these, MnO emerges as a promising system owing to its high Mn atomic ratio and spontaneous dissolution activity. In addition, composite designs like Mn_3_O_4_@nitrogen-doped carbon can also achieve high-rate performance through synergistic effects (conductive network dynamic compensation). For future research, it is imperative to combine in situ characterizations and theoretical calculations to clarify the competitive relationships of multi-mechanisms and accelerate Mn-based materials advancing toward high-energy-density, long-lifetime, and green-energy storage systems.

### 2.2. V-Based Cathodes

V-based materials are prominent candidates for aqueous zinc-ion battery cathode due to their multi-electron redox activity (V^3+^/V^4+^/V^5+^), layered/tunnel-type crystal structures, and substantial theoretical capacities. The energy storage mechanism of V-based cathodes in AZIBs fundamentally relies on their distinctive structural frameworks and multivalent redox transitions [94,95]. The specific rules followed are as follows:

(1) Zn^2+^ intercalation/deintercalation mechanism

The layered structure of vanadium oxides (e.g., V_2_O_5_ and NH_4_V_4_O_10_) offers smooth diffusion channels for Zn^2+^, while inter-layer hydrogen bonds or metal ions provide a “pillar effect”, which helps prevent structural collapse. The reaction mechanism is conventionally expressed as follows:

2V_x_O_y_ + 2nZn^2+^ + 4ne^−^ ↔ Zn_n_V_x_O_y_V_x_O_y_ + nZn^2+^ + 2ne^−^ ↔ 2Zn_n_V_x_O_y_ (16)

(2) Multi-electron redox reaction [96]

Redox reactions collectively enabled by vanadium multivalent states (V^3+^/V^4+^/V^5+^):

① First item Single-electron transition (V^5+^ ↔ V^4+^) predominates in low-voltage regions (0.5–0.8 V vs. Zn^2+^/Zn) exhibiting rapid kinetics;

② Second item Two-electron transition (V^5+^ ↔ V^3+^) occurs in high-voltage regions (0.8–1.2 V), delivering enhanced theoretical capacity.

**Figure 4:**
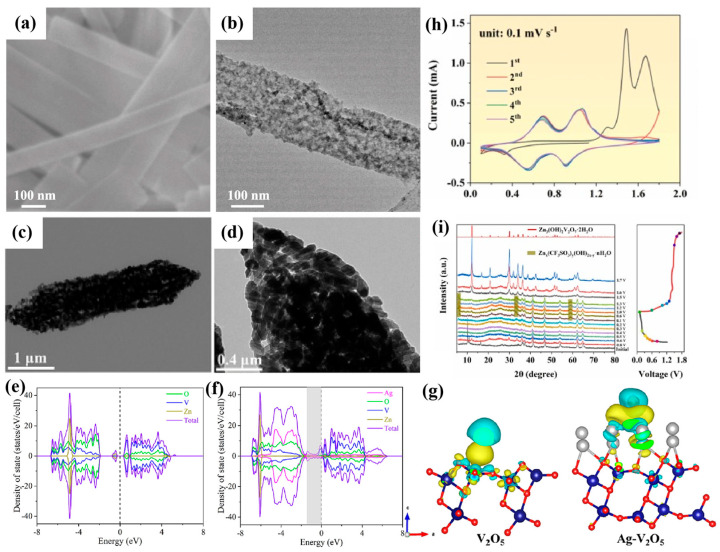
TEM images of (**a**,**b**) d-NHVO [97]; (**c**,**d**) Ag-V_2_O_5_; yotal density of states for (**e**) pristine V_2_O_5_ and (**f**) Ag-V_2_O_5_. (**g**) Differential charge density with Zn^2+^ intercalation in V_2_O_5_ and Ag-V_2_O_5_ [98]. (**h**) The first five cycles of CV curves of Mo-V-S-GO at 0.1 mV s^−1^. (**i**) XRD patterns of Mo-V-S-GO materials at different discharge/charge states in the first cycle at 0.1 A g^−1^ [109].

However, its poor intrinsic electronic conductivity and sluggish ion diffusion kinetics severely constrain its practical application. To break this limitation, Qi et al. [97] innovatively developed a room-temperature hydrazine hydrate reduction strategy to synthesize defective (NH_4_)_2_V_10_O_25_·8H_2_O nanoribbons (d-NHVO) rich in oxygen vacancies. Nanoribbons were revealed by TEM characterization to be several hundred nanometers in length and ~100 nm in width (Figure 4a,b), endowing d-NHVO with abundant active sites, superior electronic conductivity, and rapid ion diffusion kinetics. These features enable d-NHVO to deliver an exceptional capacity of 512 mAh g^−1^ at 0.3 A g^−1^ and maintain robust cycling stability with ~100% coulombic efficiency after 1000 cycles at 5 A g^−1^. In addition, to address the inherent constraints of low specific surface area and poor porosity in conventional V-based materials, Zhang et al. [98] developed an MOF-assisted topological transformation approach to fabricate hierarchical porous spindle Ag-V_2_O_5_ heterostructures as AZIB cathodes. This distinctive architecture (Figure 4c,d) confers an exceptional Zn^2+^ storage capability, showing a high reversible capacity of 426 mAh g^−1^ at 0.1 A g^−1^ and 326.1 mAh g^−1^ at 5.0 A g^−1^. After 2000 cycles, the capacity fading rate is merely 0.0053% per cycle (89.7% retention). DFT calculations (Figure 4e,f) reveal the dynamic regulatory mechanism of Ag-V_2_O_5_ heterojunctions in Zn^2+^ storage. The intercalation of Ag in the V_2_O_5_ (101) plane triggers electron reconstruction, as evidenced by a shift in the Fermi level toward the conduction band in the density distribution of electron states, which reveals the formation of high electron density states; thus, Ag-V_2_O_5_ presents metal-like conductivity. Differential charge density analysis confirms that the interfacial charge redistribution weakens the Zn-O binding energy and reduces the diffusion barriers. In the meantime, the charge transfer in Ag-V_2_O_5_ builds an electric field, which optimizes the Zn^2+^ adsorption/desorption pathways (Figure 4g). This investigation provides atomic-scale mechanistic insights to guide the design of high-rate zinc-ion batteries.

Furthermore, low conductivity and strong electrostatic interactions with Zn^2+^ of vanadium oxide will result in inadequate rate capability and poor cycling stability [99,100]. Consequently, a dual modification strategy is proposed to modulate the electronic structures: heteroatom doping via metal/non-metal incorporation [101,102,103,104,105] integrated with conductive carbon matrices (e.g., graphene, rGO/GO, CNTs) to establish three-dimensional conductive networks [106,107,108]. The cooperative approach overcomes the trade-off between energy density and cycle life of V-based materials through multiscale “electron-transport optimization/ion-diffusion acceleration/interface-stabilization” mechanisms. Li et al. [109] hydrothermally synthesized a (NH_4_)_2_(S(S_2_)Mo(S_2_))_3_/vanadium oxide/graphene oxide (Mo-V-S-GO) composite, pioneering its use as an AZIB cathode. Kinetic analysis reveals that irreversible electrochemical restructuring occurred during the initial cycling (Figure 4h), which resulted in a Mo-ion-doped vanadium heterostructure anchored on GO. Mo-doping and GO-loading synergistically enabled superior H^+^/Zn^2+^ co-insertion/extraction electrochemistry (Figure 4i), delivering 389 mAh g^−1^ at 0.5 A g^−1^. At 10 A g^−1^, it still maintained 102 mAh g^−1^ with 90.2% capacity retention after 8000 cycles. Mo-doping enhanced the V^4+^/V^5+^ redox reversibility, while GO synergistically mitigated the cyclic stress on account of the mechanical flexibility and enhanced conductivity. Such a strategy established a doping-compositing cooperative paradigm for durable zinc-ion battery cathodes.

To synergistically overcome the challenges of narrow interlayer spacing, low intrinsic conductivity, and V dissolution in V-based cathodes, Gu et al. [110] synthesized nitrogen-doped VO_2_(B) nanoribbons (VO_2_-N) through ammonia-assisted thermal treatment. Nitrogen doping induces lattice expansion and grain refinement, effectively reducing Zn^2+^ diffusion barriers, while adequate structural disorder increases active-site density through abundant grain boundaries and defects. This multiscale reconstruction optimizes ion-transport pathways and suppresses interlayer slippage by strengthening V-O/V-N bonding, achieving a high specific capacity (373.7 mAh g^−1^ at 0.1 A g^−1^) and ultralong cyclability (92.3% retention after 2000 cycles at 5 A g^−1^). This study effectively advances the high-rate and long-cycle application of V-based materials through “interlayer engineering/defect regulation” and “chemical bond reinforcement” strategies.

In summary, vanadium oxide emerges as one of the dominant cathode materials for aqueous zinc batteries owing to its multivalent redox states, tunable layered/tunnel structures, and high theoretical capacity. However, its practical application is still hindered by intrinsic limitations such as poor electronic conductivity, sluggish Zn^2+^ diffusion kinetics, narrow interlayer spacing, structural instability upon repeated cycling, and vanadium dissolution. To address these challenges, advanced modification strategies have been extensively explored. Defect engineering—such as the introduction of oxygen vacancies and heteroatom doping (e.g., N, Mo, and Ag)—effectively regulates electronic structure, expands interlayer spacing, and promotes ion transport. Additionally, composite strategies with conductive matrices (e.g., graphene and CNTs) construct robust electron transport networks and alleviate structural stress. The construction of heterointerfaces (e.g., Ag-V_2_O_5_) further induces charge redistribution and lowers diffusion barriers via built-in electric fields, thereby enhancing both rate capability and cycling stability. Through coordinated defect engineering and carbon-compositing strategies, its electronic conductivity and ion diffusion kinetics could be simultaneously enhanced. By optimizing the composition of traditional aqueous electrolytes (screening zinc salts and adding cationic additives), developing high-concentration electrolytes (salt-in-water/dual-salt systems), deep eutectic solvents, and solid/quasi-solid electrolytes, we can regulate the solvent environment, ionic composition, and interfacial reactions. This addresses issues such as cathode dissolution, sluggish ion transport, and severe side reactions, thereby enhancing battery performance [111]. In the future, research requires focused attention on heterojunction interface design, atomic-level doping optimization, and in situ characterization combined with computational analysis in multi-mechanism competition dynamics. Concurrently, developing flexible and high-energy-density devices while overcoming the bottlenecks of V dissolution and structural degradation remains essential for advancing their practical implementation.

### 2.3. Prussian Blue Analogs

Prussian blue analogs (PBAs), whose crystalline frameworks are based on metal hexacyanoferrates (MHCFs) featuring Fe-N_6_ octahedral units, show significant promise for aqueous zinc-ion batteries due to their readily available precursors, straightforward synthesis, and high operating voltages [112,113,114,115,116,117,118,119,120,121,122,123] Through the substitution of Fe atoms with transition metals (e.g., Cr, Mn, Co, or Ni) or through vacancy engineering, diverse variants with analogous composition can be acquired [114,115,116,117]. Their open three-dimensional ion channels are beneficial to facilitate reversible Zn^2+^ insertion and extraction, while their compositional tunability enables optimizing redox activity and voltage plateaus through metal-atom substitution [118,119,120,121].

However, PBA practical deployment faces significant challenges; despite theoretical capacity reaching 170 mAh g^−1^, the actual capacity typically falls below 80 mAh g^−1^ due to sluggish Zn^2+^ diffusion kinetics and insufficient active-site utilization [122]. Concurrently, structural collapse and metal dissolution (e.g., Fe^2+^ loss) induced by Jahn–Teller distortion during cycling impair long-term stability, severely hindering commercialization [123,124]. To address these issues, current research centers on multidimensional optimization. Conductive compositing (e.g., integrating PBAs with graphene/CNTs) substantially reduces interfacial charge-transfer resistance, thereby enhancing rate capability [125]; heterometal doping (e.g., Co/Ni incorporation) stabilizes crystal frameworks while broadening ion-diffusion channels [126]; nanostructuring (e.g., porous/hollow architectures) shortens ion-migration pathways to accelerate reaction kinetics [127,128]. Additionally, precise lattice-defect control (e.g., oxygen vacancies) balances Zn^2+^ adsorption dynamics and diffusion barriers, offering novel pathways to transcend capacity–cyclability trade-offs [129].

**Figure 5:**
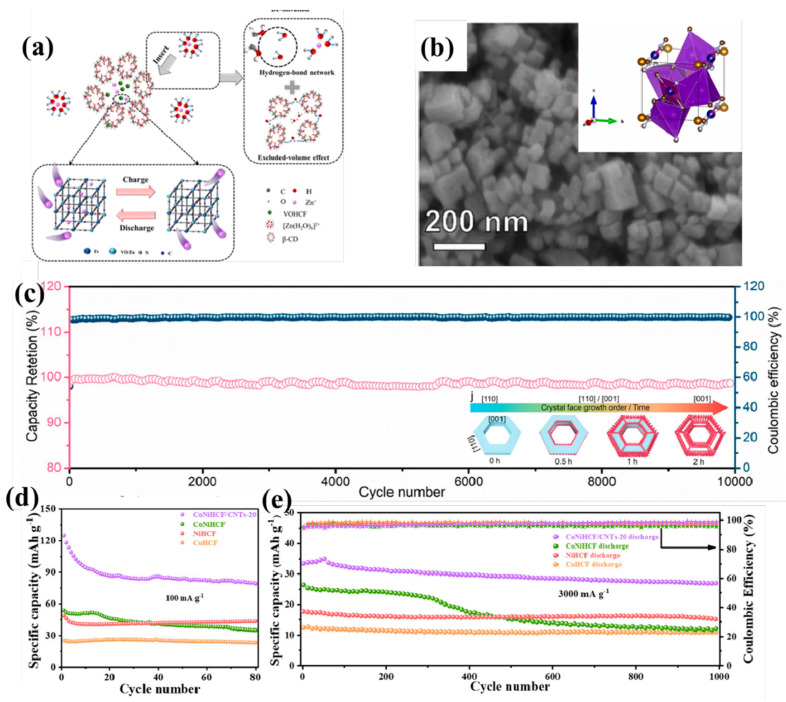
(**a**) Illustration of the charge–discharge mechanism of the β-CD-VOHCF cathode [130]; (**b**) SEM images of MnCoHCF-4, insert: schematic diagram of the crystal structure [131]; (**c**) stability test performed at 2 A g^−1^ of the DHPBA-Fe(II), with illustration of the inner–outer growth mechanism [132]. (**d**) Cycling performance at 100 mA g^−1^, (**e**) long-term cycling stability at 3000 mA g^−1^ of the CoHCF, NiHCF, CoNiHCF, and CoNiHCF/CNTs-20 electrodes [134].

Regarding heterometal doping, Hu et al. [130] incorporated multivalent V with Fe to establish dual active sites, thereby enabling multi-electron transfer and enhancing the specific capacity. Employing coprecipitation, they constructed β-cyclodextrin (β-CD)-modified vanadium hexacyanoferrate (VOHCF) featuring rich cavities and hydroxyl groups. This surface layer obstructed direct VOHCF/electrolyte contact while regulating Zn^2+^ desolvation structures, thereby improving cycling stability (Figure 5a). The resultant β-CD-VOHCF delivered high reversible capacity (204.1 mAh g^−1^ at 0.2 A g^−1^), exhibiting 65% greater capacity retention than unmodified VOHCF after 3200 cycles at 5 A g^−1^. This work establishes substantial foundations for suppressing V dissolution. In addition, Zhou et al. [131] implemented gradient cobalt substitution (partial Mn→Co replacement) in manganese hexacyanoferrate. While maintaining a consistent crystal phase and morphology (Figure 5b), the initial capacity was reduced as the Co/Mn ratio increased, but the electrochemical polarization was mitigated. The MnCoHCF-4 variant (Co/Mn = 3:1) exhibited optimal cyclability (71.4% retention after 3000 cycles at 5C) and rate performance (81.4 mAh g^−1^ at 10C). In situ analysis confirms that low Co/Mn ratios could exacerbate metal-ion dissolution, impairing structural stability and kinetics, whereas high Co content could suppress dissolution and enhance electrode robustness. This strategy offers a universal modification paradigm for high-voltage PBAs in AZIBs.

Recently, nanostructured PBAs have been designed as low-dimensional architectures and hierarchical porous frameworks to increase specific surface area and shorten ion diffusion paths, thereby significantly enhancing their electrochemical performance. Zhang et al. [132] synthesized dual-shell open-hollow PBAs—divalent iron (DHPBA-Fe(II)) and trivalent iron (DHPBA-Fe(III)) analogs—via an innovative simultaneous inward–outward growth strategy. Their formation involves lattice-matching growth and ligand exchange (Figure 5c inset). As AZIB cathodes, DHPBA-Fe(II) delivers 92.5 mAh g^−1^ at 1 A g^−1^ with exceptional 10,000-cycle stability (Figure 5c). This advances the synthesis of metal-hexacyanoferrate and elucidates the relationship between electronic structure and performance. Moreover, Ma et al. [133] employed carbothermal reduction to prepare graphene-assembled fibers, and then in situ synthesized KVO-HCF nanocrystals via a one-step liquid-phase reaction, thereby minimizing the particle size of PBA. The GSAF@KVO-HCF cathode demonstrated enhanced conductivity, prolonged cyclability (103 mAh g^−1^ after 1000 cycles at 1 A g^−1^), and superior rate performance. Cao et al. [134] pioneered cobalt–nickel hexacyanoferrate (CoNiHCF) nanocubes in situ anchored on CNTs via coprecipitation. The CoNiHCF/CNTs composite achieved 124.9 mAh g^−1^ at 50 mA g^−1^ and 81.8% capacity retention after 1000 cycles at 3000 mA g^−1^ (Figure 5d,e), which is attributed to the Co/Ni bimetallic synergism and conductive CNT scaffolding. These strategies enhance the viability of PBAs in ZIBs, expressing great potential in advancing the development of high-performance aqueous zinc-ion batteries.

Xue et al. [135] have attempted to address the inherent limitations of PBAs by constructing composite structures. For instance, recent work employs in situ co-precipitation to grow vanadyl ferricyanide (VHCF) nanoparticles onto carbon nanotubes (CNTs), significantly enhancing the electronic conductivity of the electrode material through the conductive framework provided by the CNTs. The hybrid VHCF-CNT structure not only effectively improved interparticle electronic contact but also fully utilized VHCF’s abundant ionic migration pathways, thereby substantially enhancing the reversibility of zinc-ion storage. Experimental results demonstrate that this composite material achieves a high specific capacity of 97.8 mAh g^−1^ at a current density of 50 mA g^−1^, while retaining a discharge capacity of 52.7 mAh g^−1^ after 1000 cycles at 3200 mA g^−1^. Mn-based Prussian blue analogs (Mn-based PBAs) are widely recognized as promising cathode materials for aqueous zinc-ion batteries (AZIBs) due to their high specific capacity and suitable operating potential. However, their cycling stability remains constrained by the irreversibility of ion insertion/extraction caused by structural deformation during electrochemical processes. To address this issue, B. Purusottam Reddy et al. [136] achieved significant progress through multi-metal regulation and structural optimization. For instance, one study employed PVP-assisted hydrothermal synthesis to create Mn–Co-PBA with a unique cubic framework structure. This material not only exhibited high specific surface area but also reduced structural stress during Zn^2+^ deintercalation due to lower zeolite water content within its lattice. Benefiting from this design, Mn–Co-PBA exhibited outstanding electrochemical performance: a specific capacity of 138 mAh g^−1^ at 0.1 A g^−1^, maintaining 95 mAh g^−1^ at 1 A g^−1^ and achieving a capacity retention rate of 92.8% after 1000 cycles. More importantly, in situ/post-in situ XRD analysis confirmed the highly reversible nature of its Zn^2+^ insertion/extraction process, further validating the role of the cubic framework structure in enhancing cycling stability. This study demonstrates that through rational metal synergism and structural design, Mn–Co-PBA can achieve a balance between high capacity and long lifespan, offering new insights for developing high-performance, sustainable AZIB cathode materials.

In summary, Prussian blue analogs (PBAs) represent a promising class of cathode materials for aqueous zinc-ion batteries, characterized by their open framework, tunable composition, and high operating voltage. Nevertheless, their practical application is constrained by several critical challenges, including low practical capacity (often below 80 mAh g^−1^) resulting from sluggish Zn^2+^ diffusion and insufficient active-site utilization, structural degradation due to Jahn–Teller distortion and metal dissolution (e.g., Fe^2+^), and limited cycle life. To mitigate these issues, a variety of modification strategies have been developed. Conductive compositing with carbon nanomaterials (e.g., CNTs and graphene) enhances electron transport and interfacial stability. Heterometal doping (e.g., Co, Ni, and V) strengthens structural integrity and suppresses dissolution, while also optimizing redox activity. Nanostructuring—such as the construction of hollow, porous, or low-dimensional architectures—enlarges the active surface area and shortens ion diffusion paths. Furthermore, defect engineering (e.g., oxygen vacancies or vacancy control) fine-tunes Zn^2+^ adsorption and diffusion kinetics. These multi-faceted approaches collectively contribute to improved capacity, rate performance, and cyclability, bringing PBAs closer to commercial viability. Future efforts should focus on elucidating structure–performance relationships through in situ and computational studies, refining synthetic control of defects and interfaces, and scaling the production of high-performance PBA cathodes for flexible and high-energy-density AZIB devices.

### 2.4. Organic Materials

Unlike traditional inorganic materials relying on the properties of transition metals, organic compounds primarily consist of the main-group elements (H, C, N, O, and S), exhibiting high elemental abundance and enhanced environmental compatibility. Their core advantages include high theoretical capacity derived from the reversible multi-electron redox reactions of active groups (e.g., quinone and imine); structural designability that permits precise regulation of the solubility and ion diffusion pathways through functional group modifications (e.g., sulfonic acid and carboxyl); and green sustainability owing to the accessible raw materials and mild synthesis processes. However, their practical implementation faces multiple challenges, such as energy density limitations from their low operating voltages and sluggish ion transfer kinetics; complex parasitic reactions stemming from their organic dissolution tendency and zinc-electrode interfacial reactions; and the cost–benefit imbalance caused by intricate synthesis routes for high-performance systems. Current research focuses on molecular engineering optimization and composite system design, aiming to advance organic cathode materials toward industrial application by balancing performance, lifespan, and cost synergistically.

**Figure 6:**
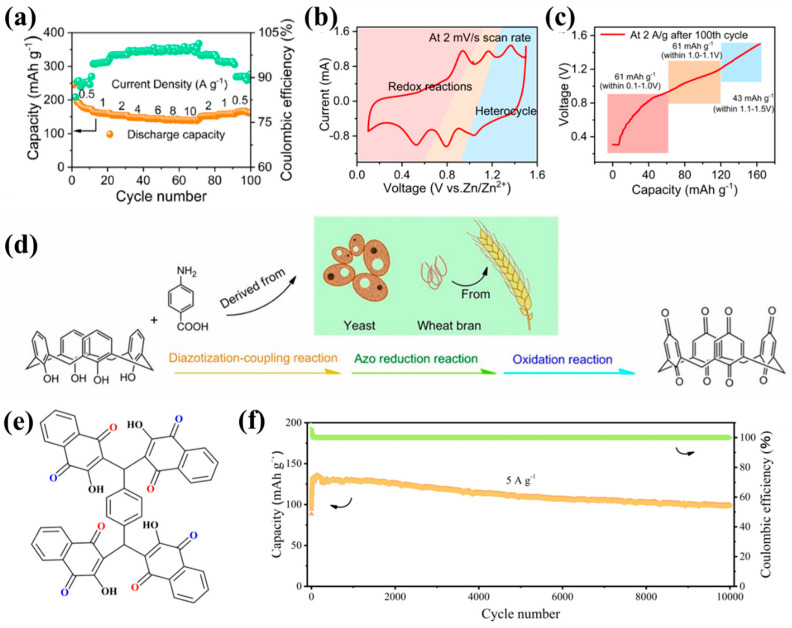
(**a**) Rate capability of the Zn||AOP coin cell with 1 M Zn(OTF)_2_ as electrolyte in the range of 0.5−10 A g^−1^. (**b**) Different voltage regions of CV for chemical reaction and coordination reaction. (**c**) Voltage vs. capacity plot at 2 A g^−1^ for 100th cycle [140]; (**d**) schematic diagram of preparing C4Q [148]; (**e**) molecular structure of TLS with two electrochemically different active sites in blue and red [149]; (**f**) cycling performance of the BPD electrode at 5 A g^−1^ [150].

Since azo compounds were first applied in lithium-ion batteries (2018) [137], their utilization in aqueous electrolytes has been restricted, due to the limited cycling stability of small molecule structures [138] and sacrificial energy density from inactive components in conventional modifications [139]. To solve these difficulties, Wang et al. [140] developed an eco-friendly diazo coupling synthesis to fabricate water-insoluble azo organic polymers (AOPs) as cathodes for RAZIBs (Rechargeable aqueous zinc batteries). The extended conjugated skeletons and crosslinked networks of AOPs deliver 170 mAh g^−1^ at 0.5 A g^−1^ (Figure 6a), maintaining 89% capacity retention after 1000 cycles at 2 A g^−1^, substantially outperforming small-molecule systems. This enhancement stems from synergistic mechanisms. The hydroxyl and azo groups present on AOPs can spontaneously form zinc-coordinated heterocyclic complexes in electrolytes, thereby contributing an additional capacity of 43 mAh g^−1^ (Figure 6b,c) and enabling dual-state Zn^2+^ storage via combined redox and coordination reactions.

Among numerous organic cathode materials, the integration of quinone and pyrazine units shows advantages in terms of high capacity and stability due to their ability to simultaneously activate multi-electron redox activity and enhance structural stability [141,142,143,144,145]. Tetrachlorobenzoquinone, a type of quinone organic compound, was applied to batteries as early as 1972. Its reduction potential in dilute H_2_SO_4_ solution is 0.7 V [146]. In recent years, quinone-based materials have been widely used in batteries, presenting exceptional stability across a broad range of pH values. In addition, across a wide temperature range and in diverse atmospheres, various ions are combined with other well-established positive electrode materials to construct stable quinone-based aqueous batteries [147]. Zhao et al. [148] studied organic molecules whose carbonyl functional groups were in ortho (such as 1,2-naphthoquinone and 9,10-phenanthrenequinone) and para (such as C4Q, 1,4-naphthoquinone, and 9,10-anthraquinone) positions. They found that para-structure can provide higher capacity when coordinating with Zn^2+^ due to its smaller steric hindrance compared to ortho-structure. Among them, C4Q has an open bowl-shaped molecular architecture (Figure 6d) containing eight carbonyl groups, and exhibits a capacity of up to 335 mAh g^−1^ at a current density of 20 mA g^−1^. However, its performance is affected by the dissolution of the positive electrode materials, resulting in rapid capacity decay and short cycle life. To address this issue, Gupta et al. [149] investigated the highly conjugated quinone molecule tetrakis lawsone (TLS) as an organic cathode material for zinc-ion batteries. The molecule is composed of four lawsone (LS) units, and its non-planar geometric structure provides sufficient space for the movement of zinc ions (Figure 6e). At a scanning rate of 0.1 mV s^−1^, the material achieved a capacity of 285 mAh g^−1^ in the first cycle and 234 mAh g^−1^ in the second cycle (87% of the theoretical capacity of 8 electrons) at 0.02 A g^−1^. After 65 cycles, the capacity decreased to 150 mAh g^−1^, with a coulombic efficiency of nearly 99%, demonstrating good electrochemical stability and reversibility. Impedance spectroscopy analysis showed that the diffusion pathway was optimized and the charge transfer resistance was reduced after cycling. Shi et al. [150] synthesized a π-conjugated nitrogen heterocyclic compound, benzo[b]phenazine-6,11-dione (BPD), which contained quinone and pyrazine redox-active functional groups. At a current density of 0.05 A g^−1^, its specific capacity could reach 429 mAh g^−1^, with a capacity utilization rate of 100%. It even exhibited good cycling performance after 10,000 cycles at a high current density of 5 A g^−1^ (Figure 6f). When the material was used as the positive electrode, the energy density of AZIBs could reach 276 Wh kg^−1^. Due to its unique molecular structure and dual redox-active functional groups, the BPD material exhibits a high specific capacity. In addition, the low water solubility of BPD and its discharge products contributes to the high efficiency and stability of the material. Therefore, various problems faced by traditional organic cathode materials of AZIBs have been effectively solved, such as limited specific capacity and utilization, unsatisfactory conductivity, poor cycle durability, and unclear charge storage mechanism. The strategy provides new ideas for the design and synthesis of high-performance organic cathode materials.

Covalent organic frameworks (COFs) are a class of porous crystalline polymer materials constructed from organic units linked by covalent bonds. Due to their high thermal stability and chemical inertness, COFs have attracted significant attention in the field of energy storage. Their core advantages lie in the designability of material structure and function. Their chemical composition, pore structure, and specific surface area can be precisely regulated through modular assembly, thereby enabling precise control of ion transport and redox behavior. Particularly, the AA stacking mode of planar COFs can form continuous two-dimensional ion diffusion channels, significantly reducing the Zn^2+^ migration barrier as well as exposing abundant active sites. Further research indicates that employing redox-active organic units (such as benzothiadiazole or triazine) to construct COF skeletons can activate multi-electron transfer reactions, endowing the materials with high specific capacity characteristics. These properties make COFs an ideal candidate cathode material for aqueous zinc-ion batteries, and their customizable molecular design provides an innovative pathway to overcome the bottlenecks of low energy density and cycle life. Artur and colleagues successfully constructed a structurally stable olefin-linked covalent organic framework, COF-TMT-BT, via aldehyde condensation between 2,4,6-trimethyl-1,3,5-triazine (TMT) and 4,4’-(benzo[c][1,2,5]thiadiazole-4,7-diyl)dibenzaldehyde (BT). This material innovatively introduced the benzothiadiazole unit as a novel electrochemically active center. Its strong electron-withdrawing effect synergistically enhanced the redox activity with the extended π-conjugated system, while the olefin linkage imparted excellent chemical stability to the framework [151]. Consequently, COF-TMT-BT, with its large pore size, provided efficient transport channels and abundant adsorption sites for Zn^2+^, resulting in a high capacity of 283.5 mAh g^−1^ at a current density of 0.1 A g^−1^.

Jaehyun Park et al. [152] demonstrated that introducing tetrabutylammonium trifluoromethanesulfonate (TBAOTf) into thermoelectric electrolytes effectively suppresses active material dissolution, thereby significantly enhancing cycling stability. Benefiting from this strategy, the Cp(CN)_6_^2−^ cathode exhibits outstanding electrochemical performance: it achieves a high discharge voltage of 1.43 V even at a high loading of 10 mg cm^−2^, retaining 85% capacity after 1000 cycles at 10C. Spectroscopic analysis further confirms that the electrochemical process involves a reversible two-electron redox reaction of Cp(CN)_6_^2−^, accompanied by the insertion/extraction of TBA^+^ ions. This achievement not only demonstrates the potential of Cp(CN)_6_^2−^ as an organic cathode based on a transformation reaction but also opens new avenues for the application of organic molecules in aqueous energy storage devices.

Organic small molecules are considered highly promising cathode materials for aqueous zinc-ion batteries (AZIBs) due to their low cost, high safety, and relatively high theoretical specific capacity. However, their widespread solubility issues severely limit cycle life and rate performance. To address this challenge, researchers have proposed strategies to reduce dissolution and expand electrochemical activity through molecular structural regulation. For instance, Hua et al. [153] recently synthesized a dihydro-octaazapentaphenanthrene (DOP) compound. Its extended N-heterocyclic structure not only significantly enhanced π-conjugation to reduce solubility but also provided dual active sites: n-type C=N groups and p-type –NH– groups that can simultaneously participate in redox reactions. Benefiting from this molecular design, the Zn//DOP battery exhibited outstanding electrochemical performance: a specific capacity of 360 mAh g^−1^ at 0.05 A g^−1^, while maintaining stability under extreme temperatures (172 mAh g^−1^ at −50 °C and 312 mAh g^−1^ at 50 °C). Further in situ and post-in situ spectroscopic analyses elucidated its energy storage mechanism, confirming that Zn^2+^/H^+^ and ClO_4_^−^ jointly participated in a multi-electron transfer process. Hua et al.’s work not only overcomes the single-ion storage limitation of conventional organic cathodes through bipolar active site design but also achieves stable operation across a wide temperature range via π-conjugated regulation. It provides critical guidance for designing high-performance, extreme-environment-tolerant organic cathodes for AZIBs, advancing zinc organic batteries toward large-scale energy storage applications.

Organic cathode materials have attracted significant attention in the field of aqueous zinc-ion batteries due to their high theoretical capacity, structural designability, and environmental friendliness. Current research focuses on systems such as quinone/azo-based compounds, conjugated polymers, and covalent organic frameworks (COFs). Molecular engineering strategies (e.g., proton coupling and π-conjugation extension) are employed to enhance redox activity and ion diffusion kinetics, achieving high capacity and long cycle life. However, their application is still constrained by low operating voltage, dissolution side reactions, and scalability costs. Future efforts should overcome the bottlenecks of low energy density and stability through the design of multi-active-site synergisms, the construction of composite conductive networks, and solid-state interface engineering. Furthermore, expansion into emerging scenarios such as flexible devices and high-entropy systems is essential to promote their practical application in high-safety, low-cost energy storage.

### 2.5. Other Types

In addition to the aforementioned materials, AZIBs also employ other widely used cathode materials with significant property advantages, such as metal–organic frameworks (MOFs) and MXenes [154,155,156,157].

**Figure 7:**
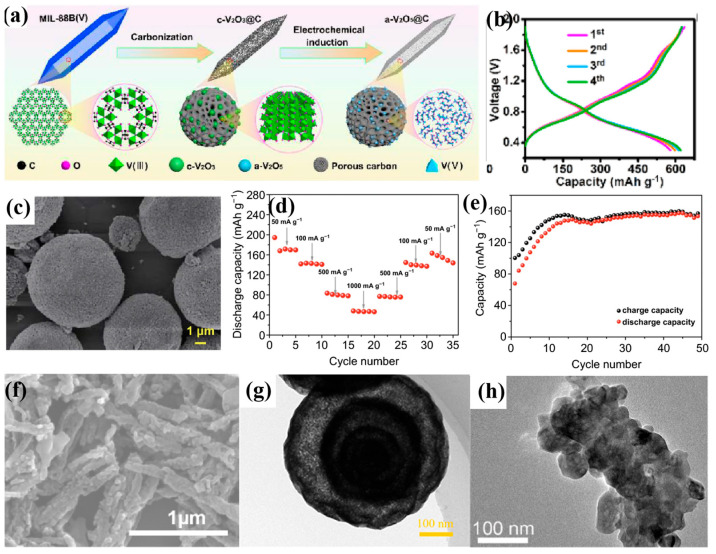
(**a**) Schematic illustration of the a-V_2_O_5_@C; (**b**) the charge/discharge curves at 0.3 A g^−1^ of a-V_2_O_5_@C [158]; (**c**) SEM image of the synthesized Mn(BTC); (**d**) rate capability and cycling performance at (**e**) at 100 mA g^−1^ in 2 M ZnSO_4_ + 0.1 M MnSO_4_ electrolyte [160]; (**f**) SEM image of α-Mn_2_O_3_ [161]; (**g**) TEM image of Mn_2_O_3_ MHS [162]; (**h**) TEM image of Mn_3_O_4_@C material [163].

V-based MOFs have emerged as promising candidate cathode materials for AZIBs due to their high specific surface area and adjustable channel structure. Their open and flexible framework provides active sites for the ordered storage of Zn^2+^ and can buffer volume strain during electrochemical processes. However, most V-based MOFs exhibit poor conductivity and structural instability in aqueous environments, resulting in unfavorable cycling performance. Therefore, V-based MOFs are often used as precursors to synthesize high-performance derived cathode materials with unique structures. For example, Deng et al. synthesized MIL-88B(V) with a pyramid-top prism morphology using a solvothermal method [158]. With MIL-88B(V) as precursor, an amorphous a-V_2_O_5_@C composite was prepared subsequently through a high-temperature calcination process. The amorphous structure endowed V_2_O_5_ with substantial isotropic Zn^2+^ diffusion pathways and active sites, resulting in rapid Zn^2+^ transport and high specific capacity. The porous carbon framework provided continuous pathways for electron transport and ion diffusion. Consequently, a-V_2_O_5_@C delivered extraordinary zinc-ion storage capability (Figure 7a,b), including an ultrahigh reversible capacity of 620.2 mAh g^−1^ at 0.3 A g^−1^, a cycling performance over 20,000 cycles at 40.0 A g^−1^ (91.4% capacity retention), and a retained capacity of 72.8 mAh g^−1^ at an ultrahigh current density of 200 A g^−1^ (≈2571 C). Zhang et al. [159] proposed introducing polyoxometalates (POMs) into metal–organic frameworks (MOFs) through confined regulation strategies to construct MOF/POM composite systems. A representative work, the Br@P-X series composites, employs a one-step solution method to precisely confine POMs within V-MOF channels, achieving synergistic structure–function optimization by adjusting the guest cluster content. This design not only significantly mitigates volume expansion in V-MOFs but also enhances electrode reaction kinetics by leveraging POMs’ superior electronic/ionic conductivity, yielding outstanding electrochemical performance. Taking Br@P-16 as an example, it demonstrates exceptional structural and chemical stability, exhibiting long-life and high-rate characteristics. More importantly, characterization techniques including X-ray Absorption Fine Structure (XAFS), in situ XRD, and XPS/FTIR revealed the structural evolution and electrochemical reaction mechanisms of the composite material during cycling. This study provides a novel approach for designing AZIB cathode materials: achieving synergistic effects between MOFs and functional clusters through confinement and interfacial regulation, thereby balancing structural stability and energy storage activity.

Mn-based MOFs and their derived materials have emerged as promising candidates for high-performance cathode materials in aqueous zinc-ion batteries owing to their porous structural characteristics and tunable ion transport channels. Xu et al. [160] systematically compared the zinc storage performance of five MOF materials: Mn(BTC), Mn(BDC), Fe(BDC), Co(BDC), and V(BDC) (BDC = 1,4-benzenedicarboxylate, BTC = 1,3,5-benzenetricarboxylate). They found that Mn(BTC) exhibited the optimal Zn^2+^ storage capability due to its unique 1,3,5-benzenetricarboxylate ligand configuration and high specific surface area. Based on this, they further investigated the properties of a full cell with Mn(BTC) as the cathode and ZIF-8@Zn as the anode, and revealed its practical application potential (Figure 7c,e). In addition, Mn-based oxides obtained through high-temperature annealing of Mn-MOFs can retain their original porous structure and morphological characteristics, thereby exhibiting excellent zinc storage performance. Mao [161] and Wang et al. [162] separately designed and synthesized two Mn-MOFs with different morphologies, which were used as templates for high-temperature annealing, resulting in Mn_2_O_3_ materials with distinct crystal structures. Specifically, Mao et al. used rod-like Mn-BTC as the precursor and obtained α-Mn_2_O_3_ after high-temperature treatment (Figure 7f). The material retains the original mesoporous structure of Mn-BTC, while the reduced specific surface area effectively inhibits the dissolution of active material, enabling a high specific capacity of 225 mAh g^−1^ at 0.05 A g^−1^ and maintaining a reversible capacity of 92.7 mAh g^−1^ after 1700 cycles at 2 A g^−1^. Comparatively, Wang et al. successfully synthesized Mn_2_O_3_ multi-layered (up to four layers) hollow nanospheres using Mn-MOF microspheres (Figure 7g). This material featured a hierarchical pore size distribution (meso-macroporous synergism), a high specific surface area (117.6 m^2^ g^−1^), and a large pore volume (0.26 cm^3^ g^−1^), endowing it with outstanding zinc-ion storage performance: a high reversible capacity of 453 mAh g^−1^ at 0.1 A g^−1^, an impressive capacity retention of 152.8 mAh g^−1^ after 500 cycles at 1 A g^−1^, and a stable output performance of 107 mAh g^−1^ at 1.5 A g^−1^. Kinetic analysis reveals that its zinc storage process was dominated by pseudocapacitive behavior, which was attributed to the multiple advantages of the hierarchical hollow structure. Firstly, the hierarchical pores promoted electrolyte infiltration and ion diffusion. Secondly, the multi-layered shell structure exposed abundant redox-active sites. Finally, the hollow cavities effectively buffered volume strain during cycling, suppressing structural pulverization. This study verified the universality of hierarchical hollow design, demonstrating that precisely controlling the topological structure of MOF-derived materials can enhance reaction kinetics and cycling stability. In research on Mn-based MOF-derived material systems, the differentiated design strategies of the structure are crucial for improving zinc-ion battery performance. Yin et al. [163] transformed an Mn-MOF precursor into a football-shaped Mn_3_O_4_@C composite via a solvothermal approach followed by an argon annealing process (Figure 7h). The uniform carbon coating not only solved the inherent poor cycling stability of Mn_3_O_4_ but also enhanced reaction kinetics through interfacial charge redistribution. As the cycle proceeded, this material exhibited unique capacity-increasing behavior at 0.5 A g^−1^, which was attributed to the gradual activation of active sites and improved electrolyte wettability. With scan rates increasing, the redox peaks of CV curves shifted but the peak shapes remained intact, indicating a dynamic balance between diffusion control and concentration polarization. This characteristic complements the pseudocapacitance-dominated multi-layered hollow Mn_2_O_3_ nanospheres previously reported by the research group led by Wang.

In recent years, electronically conductive metal–organic frameworks (EC-MOFs) have been increasingly incorporated into the cathodes and interfacial regulation of aqueous zinc-ion batteries (ZIBs) due to their combination of ordered porous structures and tunable electronic properties. Yang et al. [164] reported the application of EC-MOF DDA-Cu in ZIBs. This material not only demonstrated outstanding energy storage performance as a cathode (initial capacity of 249.6 mAh g^−1^ at 0.2 A g^−1^ and retention of 120 mAh g^−1^ after 175 cycles at 1 A g^−1^), but more importantly, its regular pore structure served as an interfacial protective layer for the zinc anode. This layer uniformly guided Zn^2+^ deposition, effectively suppressing dendrite growth and side reactions (such as hydrogen evolution reactions). Consequently, the Zn@DDA-Cu anode exhibited a stable cycling life exceeding 3500 h, while the full cell maintained a capacity of 185.5 mAh g^−1^ after 100 stable cycles at 1 A g^−1^. This study marks the first dual-functional application of conductive MOFs in ZIBs, simultaneously overcoming traditional cathode capacity and stability limitations while addressing anode dendrite challenges. It provides a critical reference for developing highly integrated, long-life ZIBs electrode materials, advancing ZIBs toward practical energy storage applications.

Due to insufficient structural stability and irreversible phase transitions, traditional cathode materials commonly face bottlenecks such as rapid specific capacity decay and limited cycle life [165]. Therefore, some special types of cathode materials have emerged recently. Among them, MXene-based two-dimensional layered materials have attracted widespread attention due to their unique multifunctional characteristics. Owing to their metal-like high conductivity, large polar surfaces, high structural tunability, and abundant active sites, MXene-based materials can simultaneously serve as cathode materials, electrolyte additives, and anode interface protective layers to synergistically regulate the redox reactions in AZIBs. Currently, Mn compounds, V compounds and organic compounds are generally used as the primary active materials to construct composite materials with MXene.

**Figure 8:**
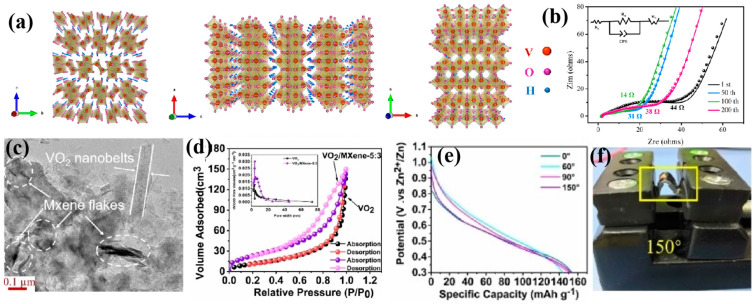
(**a**) Crystalline structure of H_2_V_3_O_8_; (**b**) Nyquist plots of the H_2_V_3_O_8_/MXene electrodes [167]; (**c**) TEM image of VO_2_/MXene-5:3; (**d**) nitrogen adsorption–desorption isotherms and pore size distribution of VO_2_ and VO_2_/MXene-5:3; (**e**) discharge curves at different bend angles of 0°, 60°, 90°, 150°, and (**f**) photograph of the flexible battery at a bend angle of 150° [168].

Layered vanadium oxides (such as H_2_V_3_O_8_), with their open layered structure and reversible Zn^2+^ intercalation characteristics, have become important cathode candidate materials for various secondary battery systems [166]. Liang et al. [167] innovatively constructed an H_2_V_3_O_8_ nanowire/MXene composite via a hydrothermal method. The presence of interlayer structural water in H_2_V_3_O_8_ (Figure 8a) can expand the interlayer spacing, providing abundant intercalation sites and rapid transmission channels for Zn^2+^. At the same time, the MXene sheets can serve as a conductive substrate, enhancing charge transport efficiency (Figure 8b). Ru et al. [168] successfully prepared a 3D VO_2_/MXene flexible film electrode through an integrative strategy combining solution mixing and freeze-drying techniques. This flexible film exhibits excellent mechanical adaptability. Its porous framework and the continuous conductive network constructed by MXene sheets provide abundant transport channels for Zn^2+^. Simultaneously, VO_2_ nanoparticles are uniformly dispersed within the MXene sheets, synergistically enhancing electrochemical activity (Figure 8c,d). Benefiting from a stable three-dimensional ion/electron dual pathway, the VO_2_/MXene cathode achieves a reversible capacity of 228.5 mAh g^−1^ at 0.2 A g^−1^ and demonstrates exceptional cycling stability. Notably, the assembled flexible zinc-ion battery maintains a stable capacity output and mechanical integrity under repeated bending conditions (Figure 8e,f), validating the application potential of this material system in flexible energy storage devices.

To address the issue of MXene stacking, Shi et al. [169] prepared high-density 3D Ti_3_C_2_T_x_@MnO_2_ via a gas-phase spray drying strategy. γ-MnO_2_ consists of randomly arranged tunnels, and its three-dimensional channels facilitate the storage of Zn^2+^. Owing to the abundant surface functional groups of Ti_3_C_2_T_x_, the 3D Ti_3_C_2_T_x_@MnO_2_ exhibits superior wettability compared to pure MnO_2_. In addition, the MXene conductive skeleton mitigates the volume expansion and dissolution of MnO_2_. The Ti_3_C_2_T_x_@MnO_2_ composite, serving as the cathode for AZIBs, delivers a reversible specific capacity of 287.3 mAh g^−1^ at 0.2 A g^−1^ and demonstrates excellent cycling stability. Furthermore, a flexible AZIB device was fabricated by coating gel electrolyte onto carbon cloth materials. This device exhibited outstanding electrochemical performance under various deformations, underscoring its significant potential for application in portable and wearable electronics.

In summary, in addition to conventional cathode systems, AZIBs have increasingly explored advanced materials such as metal–organic frameworks (MOFs) and MXenes, both offering unique structural and functional advantages yet sharing several common challenges. MOF-based cathodes, particularly V- and Mn-based frameworks, are prized for their high surface area, tunable porosity, and structural flexibility, which facilitate Zn^2+^ diffusion and accommodate volume changes. However, they often suffer from poor electronic conductivity, structural dissolution or collapse in aqueous electrolytes, and insufficient active-site accessibility, leading to rapid capacity fading and limited practical capacity. Similarly, MXene-based cathodes, though highly conductive and structurally versatile, face obstacles such as restacking of layers, oxidation instability, and challenges in integrating strongly with active host materials (e.g., metal oxides), which can compromise cycling life and rate performance.

To overcome these issues, coordinated modification strategies have been developed. For MOF-derived materials, thermal transformation into carbon-coated metal oxides helps preserve porosity while enhancing electronic conduction and inhibiting dissolution. Nanostructuring—such as designing hollow spheres, porous rods, or football-shaped architectures—increases active surface area, shortens ion diffusion paths, and alleviates mechanical strain. For MXene composites, hybridization with metal oxides leverages MXene’s conductivity and mechanical flexibility to support redox-active materials, prevent aggregation, and promote uniform distribution. The construction of 3D conductive networks—via strategies such as freeze-drying or in situ anchoring—further improves ion/electron transport and structural integrity.

These approaches collectively address universal challenges in AZIB cathodes: enhancing electrical conductivity, stabilizing electrode–electrolyte interfaces, mitigating dissolution, and controlling structural degradation. Looking forward, research should focus on the precise manipulation of interfacial properties, development of multifunctional heterostructures, and implementation of in situ/operando techniques to elucidate underlying ion storage and degradation mechanisms. Furthermore, scaling the production of tailored MOF and MXene hybrid materials and integrating them into flexible and wearable devices will be essential for advancing real-world applications of high-performance AZIBs.

## 3. Summary and Prospects

**Figure 9:**
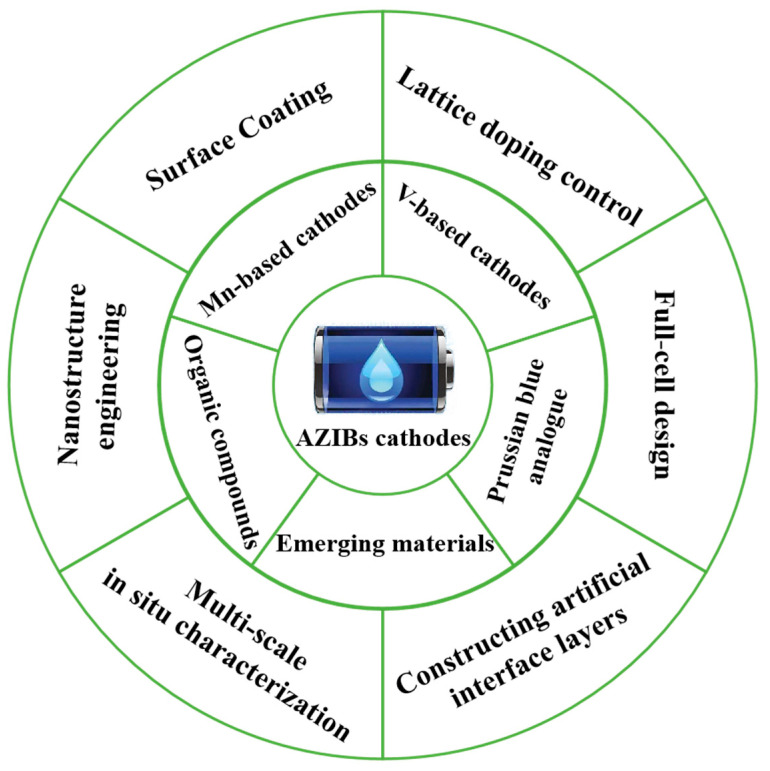
Types of AZIB cathodes and their improvement measures.

Aqueous zinc-ion batteries (AZIBs) are the next generation of highly promising energy storage systems due to their low cost, high safety, and environmental friendliness. The performance comparison of different cathode materials for aqueous zinc-ion batteries is shown in Table 1. However, their practical application still faces core challenges such as cathode dissolution, zinc dendrite growth, and low actual energy density. This article systematically reviews the research progress and existing problems of cathode materials for zinc-ion batteries. As summarized in Figure 9, which provides an overview of AZIB types and their modification strategies, the comprehensive analysis of cathode materials and future directions indicates that more efforts should be devoted to the following aspects:

(1) High performance. Focus on enhancing the actual capacity and energy density of cathode materials. Develop high-entropy composite materials, construct multi-active site structures (e.g., gradient doping and heterointerface design), and optimize dual ion/electron transport pathways to achieve high specific energy storage and fast charging/discharging (e.g., high-rate characteristics dominated by pseudocapacitive effects). Further efforts should clarify multi-ion storage mechanisms (e.g., H^+^/Zn^2+^ co-intercalation kinetics) and develop high-capacity manganese–vanadium hybrids or Prussian blue derivatives with optimized redox activity. Advanced computational screening and machine learning approaches can accelerate the discovery of novel high-voltage cathode phases.

(2) Safety enhancement. A critical direction for improving the safety of aqueous zinc-ion batteries involves suppressing intrinsic failure mechanisms—particularly the continuous dissolution of active materials (e.g., Mn^2+^ in Mn-based oxides and V in V-based compounds) and irreversible phase transitions—that contribute to capacity fading and potential internal short circuits. Mitigation strategies should focus on enhancing the structural and interfacial stability of cathode materials through ion doping, surface coating (e.g., Janus-type layers that simultaneously facilitate de-solvation and suppress parasitic reactions), and the use of high-voltage-resistant electrolytes with thermal stability. Advanced in situ/operando characterization techniques are essential to monitor real-time structural and thermal behavior under abusive conditions. Furthermore, standardized testing protocols, including nail penetration, overcharge, and thermal runaway propagation tests at the module level, should be established to evaluate and validate safety performance across different material systems, especially for V-based oxides, Mn-based oxides, Prussian blue analogs, and organic polymers. System-level design must also consider the integration of protective mechanisms to ensure operational safety under practical high-loading and high-energy-density conditions.

(3) Long lifespan. Focus on electrode-electrolyte interface engineering and the development of self-healing material systems. Achieve ultra-long cycle life exceeding 10,000 cycles by suppressing zinc dendrite growth, mitigating cathode structural collapse, and optimizing charge/discharge protocols. Deeper understanding of failure mechanisms, such as transition metal dissolution and cathode amorphization, is essential. Strategies such as lattice pinning via strong covalent bonding, pre-insertion of pillar ions, and electrolyte additives for sustained metal ion replenishment should be further explored.

(4) Environmental friendliness and sustainability. Advance the development of bio-based precursors, biodegradable polymer cathodes, and low-toxicity electrolyte systems. Establish a comprehensive life-cycle green chain encompassing raw material acquisition, production and end-of-life recycling through the incorporation of closed-loop recycling technologies. Research priorities include designing electrode materials free of critical metals, developing water-based electrode processing, and implementing energy-efficient recycling protocols such as direct regeneration and hydrometallurgical recovery.

(5) Commercialization. Accelerate deep integration of industry, academia, and research. Develop scalable processes such as continuous coating and low-temperature sintering to promote the deployment of cost-effective zinc-ion batteries in scenarios like smart grids, flexible electronics, and distributed energy storage. Simultaneously establish standardized testing systems and safety certification norms to facilitate the industrialization process. Key science-driven engineering challenges include balancing energy density with cycle life under realistic conditions, optimizing manufacturing tolerances for thick electrodes, and developing accelerated aging models to predict long-term performance.

In summary, aqueous zinc-ion batteries require coordinated breakthroughs in material innovation, mechanism understanding, and system engineering to overcome existing bottlenecks, paving the way for the practical application of high-safety, low-cost energy storage technologies.

## Figures and Tables

**Table 1:** The comparison of electrochemical performance cathodes for AZIBs.

Materials		Electrolyte	Specific Capacity (mAh/g)	Cycle Performance (% or mAh/g)	Ref.
Mn	MnO_2_/rGO	2.0 M ZnSO_4_ + 0.1 M MnSO_4_	1 A/g, 216 mAh/g	99.98%, 1.0 A/g (600 cycles)	[66]
	α-MnO_2_ (AUM)	2.0 M (ZnSO_4_) + 0.1 M (MnSO_4_)	1 A/g, 180.0 mAh/g	94.6%, 1.0 A/g (1000 cycles)	[69]
	β-MnO_2_ nanorods	1.0 M (ZnSO_4_) + MnSO_4_	0.2 A/g, 180 mAh/g	75%, 0.2 A/g (200 cycles)	[71]
	Oᵈ-MnO_2_	1.0 M (ZnSO_4_) + 0.2 mol/L M (MnSO_4_)	5 A/g, 265 mAh/g	84%, 5 A/g (2000 cycles)	[73]
	Ti-MnO_2_ NWs	3.0 M (Zn(CF_3_SO_3_)_2_) + 0.1 M (Mn(CF_3_SO_3_)_2_)	0.1 A/g, 259 mAh/g	86.9%, 0.1 A/g (200 cycles)	[75]
	ε-MnO_2_ (MnO_2_@N)	2 M (ZnSO_4_) + 0.5 M (MnSO_4_)	5 A/g, 62.5 mAh/g	83%, 5 A/g (1000 cycles)	[76]
	β-MnO_2_ (YMO)	3 M (ZnSO4) + 0.1 M (MnSO4)	0.1 A/g, 409.3 mAh/g	95.1%, 1 A/g (2000 cycles)	[77]
	α-MnO_2_@La_x_Mn_1−x_O_2−δ_ (α-MnO_2_@LMO)	2 M (ZnSO_4_) + 0.1 M (MnSO_4_)	0.5 A/g, 200 mAh/g	90%, 1 A/g (1500 cycles)	[78]
	CC@MMO	1 M (Zn(CF_3_SO_3_)_2_) + 0.1 M (MnSO_4_)	0.1 A/g, 170.2 mAh/g	80%, 0.5 A/g (1392 cycles)	[79]
	PVP-Al-MnO_2_	3 M (Zn (ClO_4_)_2_) + 0.1 M (MnSO_4_)	0.3 A/g, 306.8 mAh/g	93.1%, 1.0 A/g (2000 cycles)	[80]
	GQDs@ZnₓMnO_2_	1 M (ZnSO_4_)	0.3 A/g, 403.6 mAh/g	88.1%, 1.0 A/g (500 cycles)	[84]
	WBEC	3 M (ZnSO_4_) + 0.1 M (MnSO_4_)	0.1 A/g, 312 mAh/g	80.1%, 1.0 A/g (1000 cycles)	[86]
	Cu/MnOₓ	2 M (ZnSO_4_)	0.2 A/g, 304.4 mAh/g	76.6%, 0.5 A/g (1000 cycles)	[87]
	MnO/C-PDA	2 M (ZnSO_4_) + 0.2 M (MnSO_4_)	0.1 A/g, 295.4 mAh/g	88.9%, 1.0 A/g (500 cycles)	[88]
	MnO@CNF-1	0.3 M (MnSO_4_) + 2.0 M (ZnSO_4_)	0.1 A/g, 392 mAh/g	110%, 2.0 A/g (1800 cycles)	[90]
V	d-(NH_4_)_2_V_10_O_25_·8H_2_O	3 M (Zn (CF_3_SO_3_)_2_)	0.3 A/g, 512 mAh/g	90%, 5 A/g (1000 cycles)	[97]
	Ag-V_2_O_5_	3 M (Zn (CF_3_SO_3_)_2_)	0.1 A/g, 426 mAh/g	89.7%, 5 A/g (2000 cycles)	[98]
	ZnVOH/CC	2 M (ZnSO_4_)	0.5 A/g, 200 mAh/g	100%, 20 A/g (5000 cycles)	[99]
	Na_6_V_10_O_28_	2 M (ZnSO_4_)	0.1 A/g, 320 mAh/g	90%, 2 A/g (1000 cycles)	[101]
	Cs_0_._5_V_2_O_5_	3 M (Zn (CF_3_SO_3_)_2_)	0.1 A/g, 380 mAh/g	90%, 5 A/g (2000 cycles)	[102]
	(NH_4_)_2_V_10_O_25_·8H_2_O	3 M (Zn (CF_3_SO_3_)_2_)	0.1 A/g, 408 mAh/g	94.1%, 5 A/g (4000 cycles)	[103]
	Ni_0_._011_V_0_._989_O_2_	3 M (Zn (CF_3_SO_4_)_2_)	0.1 A/g, 295.9 mAh/g	88.9%, 10 A/g (2000 cycles)	[104]
	Zn_0_._25_V_2_O_5_·nH_2_O	3 M (Zn (CF_3_SO_3_)_2_)	0.1 A/g, 209.6 mAh/g	80.7%, 5 A/g (10,000 cycles)	[105]
	Mn-d-ZMO@C	2 M (ZnSO_4_) + 0.2 M (MnSO_4_)	0.1 A/g, 194 mAh/g	84%, 3 A/g (2000 cycles)	[106]
PBA	Na_1.88_Fe[Fe(CN)_6_]_0.840.16_·3.11H_2_O	1 M NaClO_4_/PC-FEC	0.1 C, 140 mAh/g	83%, 0.5 C (100 cycles)	[112]
	FeCu–PB@CuO	1 M NaClO_4_/PC-FEC	0.1 A/g, 123.5 mAh/g	75.4%, 1.0 A/g (1000 cycles)	[118]
	Na_2−x_MnFe (CN)_6_ (MnHCF-S-170)	1 mol/L NaClO EC/DEC (1:1, *v*/*v*)	10 mA/g, 164 mAh/g	57.1%, 0.1 A/g (500 cycles)	[119]
	MnHCF	1 mol/L NaPF_6_EC/DEC (1:1, *v*/*v*)	0.1 C, 121.9 mAh/g	65%, 0.2 C (100 cycles)	[122]
	MnHCF@PEDOT-20	1 mol/L NaPF_6_EC/DEC (1:1, *v*/*v*)	15 mA/g, 147.9 mAh/g	78.2%, 10 C (1000 cycles)	[123]
	K_1_._94_Mn [Fe (CN)_6_]_0_._994_·0.08H_2_O	3 M (KFSI) (TEP)/EC/DEC (1:1, *v*/*v*)	0.1 C, 140–155 mAh/g	80%, 5 C (5800 cycles)	[124]
	NiHCF/RGO	2 M (ZnSO_4_)	5 mA/g, 94.5 mAh/g	80.3%, 4 C (1000 cycles)	[125]
	CoMn-PBA HSs	2 M (ZnSO_4_)	0.05 A/g, 128.6 mAh/g	76.4%, 1 A/g (1000 cycles)	[128]
	MnCoHCF-4	1 M (Zn(CF_3_SO_3_)_2_) + 2 M (LiTFSI)	1 C, 101.3 mAh/g	71.4%, 5 C (3000 cycles)	[131]
	DHPBA-Fe (II)	1 M (ZnSO_4_)	1 A/g, 92.5 mAh/g	99.2%, 2 A/g (1000 cycles)	[132]
	GSAF@KVO-HCF	2 M (ZnSO_4_)	0.1 A/g, 162 mAh/g	86.6%, 1 A/g (1000 cycles)	[133]
	VHCF/CNTs	2 mol/L (ZnSO_4_)	50 mA/g, 97.8 mAh/g	53.9%, 3200 mA/g (1000 cycles)	[135]
	Mn-Co-PBA@CF	3 mol/L (Zn (OTf)_2_)	0.1 A/g, 138 mAh/g	92.8%, 0.5 A/g (1000 cycles)	[136]
Organic materials	AOPs	1 M (Zn (OTF)_2_)	0.5 A/g, 170 mAh/g	89%, 2 A/g (1000 cycles)	[140]
	HATNQ	3 M (ZnSO_4_)	0.2 A/g, 482.5 mAh/g	99.99%, 5 A/g (11,000 cycles)	[141]
	HAQ-COF	2 M (ZnSO_4_)	0.1 A/g, 344 mAh/g	85%, 5 A/g (10,000 cycles)	[143]
	TTPQ	2 M (ZnSO_4_)	0.3 A/g, 404 mAh/g	94%, 0.5 A/g (250 cycles)	[144]
	TDT	1 M (ZnSO_4_)	0.2 A/g, 369 mAh/g	75.6%, 0.2 A/g (200 cycles)	[145]
	BPD	2 M (ZnSO_4_)	0.05 A/g, 429 mAh/g	73%, 5 A/g (10,000 cycles)	[150]
	COF-TMT-BT	2 M (Zn (CF_3_SO_3_)_2_)	0.1 A/g, 283.5 mAh/g	65.9%, 0.1 A/g (2000 cycles)	[151]
	TBA_2_Cp (CN)_6_	1 mol/L (Zn (OTf)_2_) + 30 mM (TBAOTf)	0.05 C, 78.5 mAh/g	85%, 10 C (1000 cycles)	[152]
	DOP	2 mol/L (Zn (ClO_4_)_2_)	0.05 A/g, 360 mAh/g	93.3%, 0.2 A/g (500 cycles)	[153]
	Br@P-16	3 mol/L (Zn (CF_3_SO_3_)_2_)	0.2 A/g, 151.9 mAh/g	62.9%, 3.0 A/g (2500 cycles)	[158]
Other types	a-V_2_O_5_@C	2 M (Zn (CF_3_SO_3_)_2_)	0.3 A/g, 620.2 mAh/g	91.4%, 40 A/g (20,000 cycles)	[159]
	α-Mn_2_O_3_	2 M (ZnSO_4_) + 0.2 M (MnSO_4_)	0.05 A/g, 225 mAh/g	53.3%, 2 A/g (1700 cycles)	[161]
	Mn_2_O_3_ MHS	2 M (ZnSO_4_) + 0.1 M (MnSO_4_)	0.1 A/g, 453 mAh/g	98.7%, 1 A/g (500 cycles)	[162]
	DDA-Cu	3.5 M Zn(CF_3_SO_3_)_2_	0.2 A/g, 249.6 mAh/g	—, 2 A/g (100 cycles)	[164]
	H_2_V_3_O_8_/MXene	3 M (Zn (CF_3_SO_3_)_2_)	0.1 A/g, 428 mAh/g	76.9%, 10 A/g (9000 cycles)	[167]
	VO_2_/MXene-5:3	2 M (Zn (CF_3_SO_3_)_2_)	0.2 A/g, 228.5 mAh/g	80%, 2 A/g (700 cycles)	[168]
	3D Ti_3_C_2_Tₓ@MnO_2_	2 M (ZnSO_4_) + 0.1 M (MnSO_4_)	100 mA/g, 301.2 mAh/g	90.6%, 500 mA/g (2000 cycles)	[169]

## Data Availability

No new data were created or analyzed in this study. Data sharing is not applicable to this article.
